# Retrospective Cohort Study: Scope for Improvement—Barriers to Post‐Polypectomy Surveillance in the Integrated Technologies for Improved Polyp Surveillance Cohort

**DOI:** 10.1111/apt.18514

**Published:** 2025-01-20

**Authors:** Charlotte Mathews, Aleena Nauman, Mark Johnstone, Reiss Stoops, Alexander Tham, Emma C. Parsons, Kathryn A. Robb, William Sloan, Gerard Lynch, Joanne Edwards, Stephen T. McSorley, the INCISE collaborative group

**Affiliations:** ^1^ School of Cancer Sciences, College of Medical Veterinary and Life Sciences University of Glasgow, Wolfson Wohl Cancer Research Centre, Garscube Estate Glasgow UK; ^2^ Academic Unit of Surgery Glasgow Royal Infirmary Glasgow UK; ^3^ School of Health and Wellbeing College of Medical Veterinary and Life Sciences Glasgow UK; ^4^ NHS Greater Glasgow and Clyde, Research and Innovation Queen Elizabeth University Hospital Glasgow UK

**Keywords:** adenoma, colorectal, deprivation, polyp, surveillance

## Abstract

**Background:**

Adherence to post‐polypectomy surveillance is poor despite evidence that it is associated with lower risk of future colorectal cancer.

**Methods:**

We evaluated 6,210 bowel screening participants between 2009‐2016 in NHS Greater Glasgow and Clyde to assess potential barriers to post‐polypectomy surveillance.

**Results:**

Increasing deprivation (Scottish Index of Multiple Deprivation quintile 1 vs 5; OR 1.68; *p* < 0.001), and increasing comorbidity (Charlson Comorbidity Index 1‐2 vs 3‐4; OR 1.80; *p* < 0.001, vs ≥ 5; OR 3.31; *p* < 0.001), were associated with non‐surveillance in British Society of Gastroenterology 2002 intermediate/high‐risk patients, while ACE‐Inhibitor (OR 0.78; *p* < 0.001) and aspirin use (OR 0.34; *p* < 0.001) were associated with undergoing surveillance. The most deprived patients receiving surveillance had more metachronous polyps (54.0% vs 49.3%) and cancer (1.1% vs 0.4%) (*p* = 0.044).

**Discussion:**

Patients from more socioeconomically deprived areas are less likely to have appropriate post‐polypectomy surveillance, and are more likely to have metachronous polyps and colorectal cancer even when they do.

**Conclusion:**

Surveillance strategies must take into account factors including socioeconomic deprivation and comorbidity exist to improve surveillance uptake in this group through the design of targeted interventions which move away from the current “one size fits all” approach.

## Graphical abstract

Within 6210 bowel screening participants between 2009 and 2016 in NHS Greater Glasgow and Clyde, increasing deprivation (Scottish Index of Multiple Deprivation quintile 1 vs. 5); was associated with non‐surveillance (OR 1.68; *p* < 0.001), and more metachronous polyps (54.0% vs. 49.3%) and cancer (1.1% vs. 0.4%) (*p* = 0.044) in those who were surveyed.

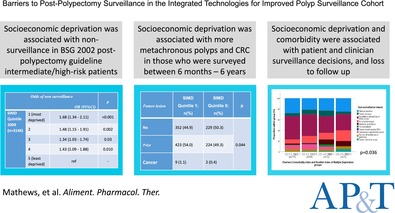

## 1 Introduction

Despite guidelines being accurate in identifying risk for future colorectal cancer (CRC), adherence to post‐polypectomy surveillance is suboptimal within the UK and worldwide [1, 2, 3]. Post‐polypectomy surveillance uptake is thought to be multifactorial at the patient, provider and healthcare system levels. An American study illustrated that age, sex, comorbidities, socioeconomic deprivation, cancer prognosis, ethnicity and insurance status affect cancer surveillance uptake [4]. However, this study was from a healthcare system substantially different to that of the UK; since only insured patients were recruited, a considerable proportion of the population were excluded [4].

This observational study aimed to compare patient and polyp characteristics of patients classified as intermediate or high risk using British Society of Gastroenterology (BSG) 2002 criteria [5], who did and did not receive surveillance colonoscopy within 6 years of their index screening polypectomy. Furthermore, we aimed to examine those who had deviated from the BSG guidance and explore the potential reasons and barriers to surveillance.

## 2 Methods

### 2.1 Patient Cohort

**FIGURE 1:**
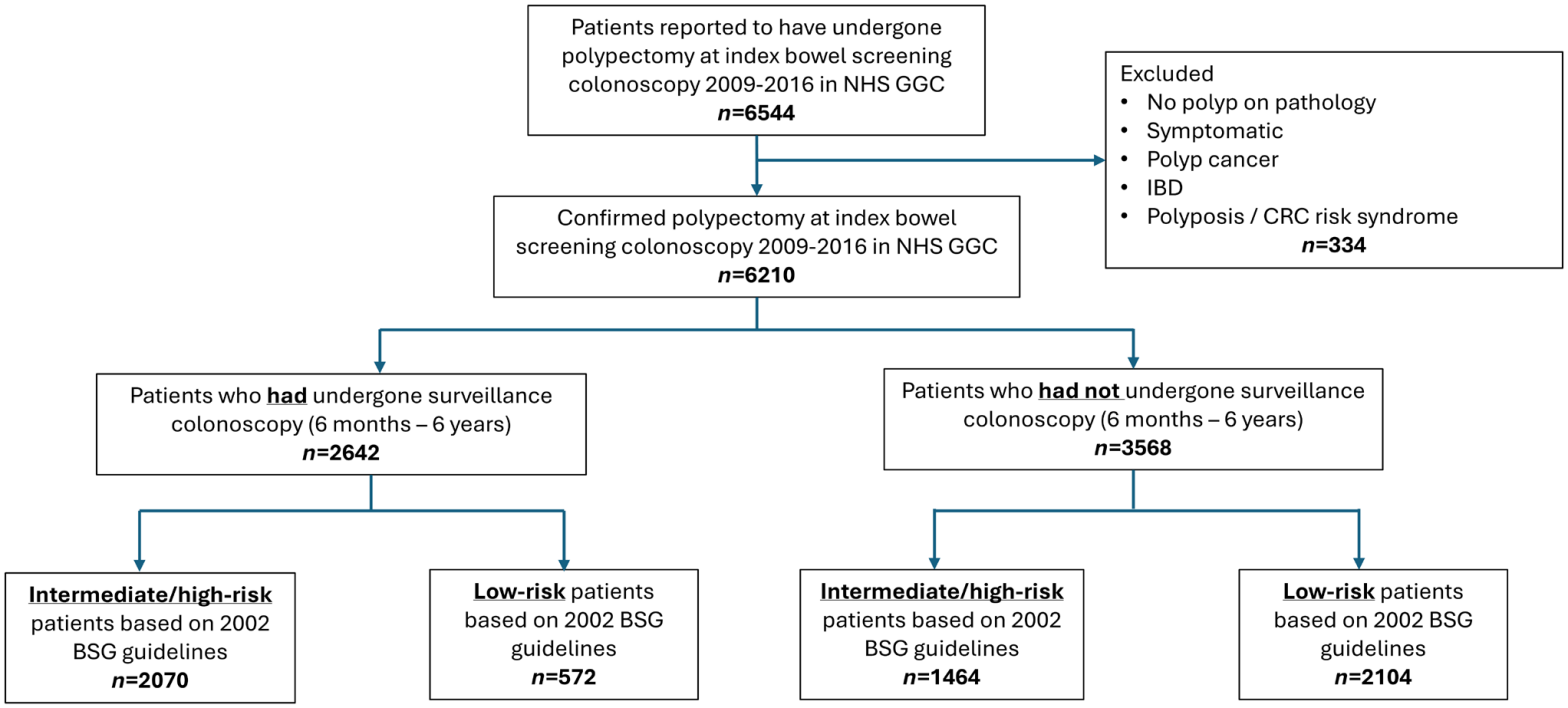
Flow diagram outlining the Integrated Technologies for Improved Polyp Surveillance cohorts based on surveillance and risk stratification. BSG, British Society of Gastroenterology Guidelines; CRC, colorectal cancer; IBD, inflammatory bowel disease; NHS GGC, National Health Service Greater Glasgow and Clyde.

A database of patients undergoing polypectomy at colonoscopy within the Scottish Bowel Screening Programme (SBSP) in NHS Greater Glasgow and Clyde (NHC GGC) from May 2009 until December 2016 led to the creation of the INCISE cohort, divided into those who had and had not received a subsequent colonoscopy between 6 months and 6 years after initial screening colonoscopy (Figure 1) [6].

We excluded patients with no polyps at index colonoscopy, polypectomy through the symptomatic pathway, with CRC at screening colonoscopy, with polyposis or inherited CRC syndromes, and inflammatory bowel diseases. Each patient was allocated a distinctive INCISE number linked to the unique national Community Health Index (CHI) identifier and data were held within the NHS Glasgow SafeHaven TRE platform to ensure anonymity and data protection. Ethical approval was obtained (GSH/20/CO/002) and outcomes were reported in accordance with the STROBE guidelines.

Data were collected from electronic clinical records (Clinical Portal), endoscopy reporting software (Unisoft Medical Systems GI Reporting Software Version 22) and electronic pathology databases (TelePath) linked by CHI.

### 2.2 Outcomes of Interest

The primary outcome was non‐surveillance in patients categorised as intermediate/high‐risk using BSG 2002 guidelines. Associations with patient, clinical and pathological features were investigated by comparing non‐surveyed intermediate/high‐risk patients with surveyed intermediate/high‐risk patients. Non surveyed patients were investigated to capture potential reasons from electronic case notes.

### 2.3 Included Variables and Statistical Analysis

Univariate analysis of categorical variables including sex, medications, co‐morbidities, index polyp features (advanced, histology, and size), index polyp location (right colon: caecum to splenic flexure, left colon: proximal descending to distal sigmoid), and BSG 2002 future risk criteria, used chi‐squared (*X* ^2^) tests and linear by linear association. Scottish Index of Multiple Deprivation (SIMD) 2009 was assessed by grouping patients into post code‐based quintiles assigned to the entire country, with the first quintile being most deprived. We compared medians of continuous variables by Mann–Whitney *U*‐test. Multivariate binary logistic regression (backward conditional model) assessed independence of association with the dependent variable non‐surveillance for variables significant at univariate analysis. Missing data were excluded on a variable by variable basis at univariate analysis and listwise at multivariate analysis. Statistical significance was defined as a value of *p* < 0.05. IBM Statistical Package for the Social Sciences version 22 was used for all analyses.

## 3 Results

### 3.1 Study Population

From May 2009 until December 2016, 6210 patients underwent index polypectomy within SBSP in NHS GGC (Figure 1). Following BSG 2002 guidelines, 2676 patients (43%) were low‐risk, 2353 (38%) intermediate‐risk and 1181 (19%) high‐risk.

### 3.2 Co‐Morbidity and Socioeconomic Deprivation Are Associated With Non‐surveillance in BSG 2002 Intermediate and High‐Risk Groups

Of all intermediate/high‐risk patients, 2070 (58%) underwent surveillance and 1464 (42%) did not (Table 1). Intermediate/high‐risk patients who were not surveyed were more likely to live in areas of socioeconomic deprivation (*p* < 0.001) with a higher burden of co‐morbidities (*p* < 0.001), but were less likely to be prescribed medications such as angiotensin converting enyme inhibitors (ACE‐I), angiotensin receptor blockers (ARB), aspirin and statins (all *p* < 0.001). They were more likely to have single and smaller polyps (both *p* < 0.001) and serrated histology (*p* < 0.001) despite being intermediate or high‐risk. At multivariate analysis, SIMD 1; OR 1.68 (95% CI, 1.34–2.11), 2; OR 1.48 (95% CI, 1.15–1.91), 3; OR 1.34 (95% CI, 1.03–1.74), 4; OR 1.43 (95% CI, 1.09–1.88), CCI (3–4; OR 1.80 (95% CI, 1.52–2.14), ≥ 5; OR 3.31 (95% CI, 2.52–4.34)), and serrated histology (OR 1.96 (95% CI, 1.42–2.69)) were independently associated with non‐surveillance, while ACE‐I (OR 0.78 *p* = 0.011), ARB (OR 0.59 *p* < 0.001), aspirin (OR 0.34 *p* < 0.001) and polyp characteristics advanced index polyp (OR 0.71 (95% CI 0.58–0.87)), polyp number (2–4; OR 0.69 (95% CI, 0.58–0.82), ≥ 5; OR 0.51 (95% CI, 0.40–0.66)) were associated with having surveillance in intermediate/high‐risk patients.

**TABLE 1:** Comparison of patient demographics, co‐morbidities, medications and polyp characteristics between intermediate/high‐risk patients who did and did not receive surveillance post‐polypectomy based on the 2002 British Society of Gastroenterology Guidelines.

		All (*n* = 3534) (%)	Intermediate/High risk with surveillance; *n* = 2070 (%)	Intermediate/High risk with no surveillance; *n* = 1464 (%)	*p*	Multivariate OR for non‐surveillance (95% CI)	*p*
Demographics (*n* = 3534)							
Age (years)	Median (IQR)	63 (57–69)	63 (57–69)	63 (57–69)	0.93	—	—
Sex	Female	1069 (30)	606 (29)	463 (32)	0.13	—	—
	Male	2465 (70)	1464 (71)	1001 (68)		—	—
SIMD Quintile 2009 (*n* = 3146)	1 (most deprived)	1088 (35)	612 (33)	476 (37)	< 0.001	1.68 (1.34–2.11)	< 0.001
	2	564 (18)	323 (17)	241 (19)		1.48 (1.15–1.91)	0.002
	3	511 (16)	306 (16)	205 (16)		1.34 (1.03–1.74)	0.03
	4	434 (14)	255 (14)	179 (14)		1.43 (1.09–1.88)	0.010
	5 (least deprived)	549 (17)	366 (20)	183 (14)		ref	—
Co‐morbidities (*n* = 3534)							
CCI	1–2 (fewest co‐morbidities)	2108 (60)	1314 (64)	794 (54)	< 0.001	ref	—
	3–4	1091 (31)	607 (29)	484 (33)		1.80 (1.52–2.14)	< 0.001
	≥ 5 (most co‐morbidities)	335 (9)	149 (7)	186 (13)		3.31 (2.52–4.34)	< 0.001
Medication (*n* = 3534)							
ACE‐I	Yes	802 (23)	532 (26)	270 (18)	< 0.001	0.78 (0.64–0.94)	0.01
	No	2732 (77)	1538 (74)	1194 (82)		ref	—
ARB	Yes	330 (9)	231 (11)	99 (7)	< 0.001	0.59 (0.45–0.79)	< 0.001
	No	3204 (91)	1839 (89)	1365 (93)		ref	—
Aspirin	Yes	809 (23)	604 (29)	205 (14)	< 0.001	0.34 (0.28–0.42)	< 0.001
	No	2725 (77)	1466 (71)	1259 (86)		ref	—
Statin	Yes	1377 (39)	874 (42)	503 (34)	< 0.001	0.99 (0.82–1.20)	0.91
	No	2157 (61)	1196 (58)	961 (66)		ref	—
Pathology (*n* = 3534)							
Advanced index polyp	Advanced	2915 (83)	1741 (84)	1174 (80)	0.003	0.71 (0.58–0.87)	0.001
	Non‐advanced	619 (17)	329 (16)	290 (20)		ref	—
Index polyp number	1	1041 (30)	557 (27)	484 (33)	< 0.001	ref	
	2–4	1979 (56)	1179 (57.0)	800 (55)		0.69 (0.58–0.82)	< 0.001
	≥ 5	514 (14)	334 (16)	180 (12)		0.51 (0.40–0.66)	< 0.001
Index polyp type	Adenoma	3332 (94)	1984 (96)	1348 (92)	< 0.001	ref	
	Serrated	201 (6)	86 (4)	115 (8)		1.96 (1.42–2.70)	< 0.001
Index polyp size	< 10 mm	632 (18)	342 (17)	290 (20)	0.012		
	≥ 10 mm	2902 (82)	1728 (84)	1174 (80)		1.20 (0.63–2.31)	0.58
Index polyp location^a^	Right colon	787 (22)	461 (22)	326 (22)	0.13	—	—
	Left colon	2248 (64)	1343 (65)	905 (62)		—	—
	Rectum	489 (14)	260 (13)	229 (16)		—	—

*Note:* Comparison between baseline characteristics between intermediate/high‐risk surveillance and non‐surveillance patients were statistically analysed by the Mann–Whitney *U*‐test for continuous variables, *X* ^2^ test for categorical variables and *X* ^2^ for linear trends for further ordered categorical variables. SIMD quintile 1 refers to the most deprived groups versus quintile 5 which refers to the least deprived.

Abbreviations: ACE‐I, angiotensin‐converting enzyme inhibitors; ARBs, angiotensin receptor blockers; CCI, Charlson Co‐morbidity Index; CI, confidence interval; COPD, Chronic Obstructive Inflammatory Disease; IQR, Inter‐quartile range; OR, Odds Ratio; SIMD, Scottish Index of Multiple Deprivation.

^a^ Right colon, proximal to splenic flexure; Left colon, splenic flexure to rectosigmoid junction; Rectum, rectum—all as recorded by the endoscopist on the screening colonoscopy report.

### 3.3 Socioeconomic Deprivation Is Associated With Higher Risk of Future Polyp or CRC in Those Patients Who Do Undergo Post‐Polypectomy Surveillance

Comparison of those who underwent surveillance in SIMD quintile 1 (most deprived, *n* = 784) to 5 (least deprived, *n* = 455), found a significantly higher rate (*p* = 0.044) of future polyps (54% vs. 49%) and cancer (1.1% vs. 0.4%), despite quintile 1 having fewer advanced adenomas at index colonoscopy (62% vs. 72%, *p* < 0.001).

### 3.4 Reasons for Deviation From the BSG 2002 Post‐Polypectomy Surveillance Guidance

Within the intermediate/high‐risk group 352 (24%) patients were advised against surveillance by clinicians, 131 (9%) chose not to have surveillance, and 101 (7%) underwent surveillance outside the 6 month–6 year interval (Figure S1). Furthermore, 556 (39%) were advised to have a surveillance colonoscopy but did not, 203 (14%) had no recorded reason, 56 (4%) died before surveillance, and 0.3% moved outside the NHS GGC board area. Surveillance was also altered by the guideline changed in 2020 leading to patient removal from waiting lists (2.7%). When patients were grouped by extremes of CCI and SIMD, there were small but statistically significant differences (*p* = 0.036) in reasons attributed for non‐ surveillance. Co‐morbidity was particularly associated with non‐surveillance despite clinician recommendation; deprivation was associated with clinician decision against surveillance.

## 4 Discussion

This study synthesises demographic, clinical, histological and endoscopic data in screened polypectomy patients, followed from the index procedure through to surveillance outcome. Of those categorised intermediate‐ or high‐risk using the BSG 2002 guidelines, 41% did not undergo a surveillance/subsequent colonoscopy within 6 years of their index bowel screening polypectomy. Higher levels of socioeconomic deprivation were significantly and independently associated with non‐surveillance, even in high‐risk groups, and were also associated with higher risk of future polyp or CRC in those who underwent surveillance colonoscopy.

Literature on cancer and post‐polypectomy surveillance suggests that deprivation is associated with reduced surveillance [4, 6]. Nearly half of the NHS GGC population lives within the most deprived 20% of areas in Scotland. This health board also has the lowest uptake of screening in Scotland (62%). Studies have shown inequality among bowel cancer screening uptake in hose living in highly deprived areas, with multiple driving factors [7]. Reduced health literacy can be associated with educational attainment, which may impact understanding of screening and surveillance, and impact health care interactions [8]. In addition, those from areas of socioeconomic deprivation may face financial barriers to surveillance, reduced access to primary and secondary care, and difficulty attending appointments due to logistical and infrastructure issues [8]. Furthermore, those from areas of socioeconomic deprivation are more likely to be from ethnically diverse groups with cultural/social factors impacting surveillance and the potential for distrust of government and healthcare systems [8]. Finally, those from more deprived backgrounds have a higher prevalence of co‐morbidity with competing healthcare needs reducing the priority of surveillance [8].

Prescribed cardiovascular secondary prevention medications, associated with metachronous lesion risk [9], were associated with undergoing surveillance, while higher CCI was associated with non‐surveillance. Further analysis reveals that the prescription of aspirin, ACE‐Is and ARBs was associated with increasing CCI. However, within each CCI subgroup, it was higher in those who had surveillance (Figure S2). This discrepancy may relate to patients' health beliefs and initial screening participation which may influence subsequent surveillance choices and broader engagement with healthcare, or positive pro‐surveillance and screening interaction with primary care physicians, which we did not capture.

Further limitations are the inclusion only of patients entering surveillance through bowel screening. The use of SIMD as an area‐based measure of deprivation, which may lead to misclassification of individuals within a given postcode, with the pre‐specified domains only proxy measures of socioeconomic deprivation. Community prescribing data did not take into account length of prescription prior to index screening colonoscopy.

The finding that patients from more socioeconomically deprived areas are less likely to have appropriate surveillance, and have poorer outcomes even when they do, is important and suggests that surveillance strategies need to be tailored to such groups. Evidence‐based frameworks for improving screening and surveillance behaviour, take into account factors including socioeconomic deprivation [10]. In future, they may be utilised to improve surveillance uptake in this group through the design of targeted interventions which move away from the current ‘one size fits all’ approach.

## Author Contributions

**Charlotte Mathews:** data curation, formal analysis, visualization, methodology, investigation, writing – original draft. **Aleena Nauman:** data curation, formal analysis, visualization, writing – original draft, methodology, investigation. **Mark Johnstone:** data curation, investigation, methodology, resources, software, writing – review and editing. **Reiss Stoops:** data curation, investigation, writing – review and editing. **Alexander Tham:** data curation, investigation, writing – review and editing. **Emma C. Parsons:** methodology, project administration, writing – review and editing. **Kathryn A. Robb:** supervision, writing – review and editing, methodology. **William Sloan:** data curation, methodology, resources, software. **Gerard Lynch:** conceptualization, funding acquisition, supervision, writing – review and editing. **Joanne Edwards:** conceptualization, funding acquisition, supervision, project administration, writing – review and editing. **Stephen T. McSorley:** conceptualization, data curation, formal analysis, investigation, methodology, project administration, resources, software, supervision, writing – original draft, writing – review and editing, funding acquisition, validation.

## Ethics Statement

Ethical approval was obtained (GSH/20/CO/002) and outcomes were reported in accordance with the STROBE guidelines.

## Consent

The authors have nothing to report.

## Conflicts of Interest

The authors declare no conflicts of interest.

## Supporting information


**Figure S1:** (A) Histogram describing reasons for non‐surveillance in those intermediate/high risk patients not following the 2002 British Society of Gastroenterology Guidelines (*n* = 1446). (B) Stackplot presenting categorised reasons for non‐surveillance among intermediate/high risk patients in the extremes of Charlson Comorbidity Index (CCI 1–2 = least comorbid, CCI > =5 most comorbid) and Scottish Index of Multiple Deprivation (SIMD 1 = most deprived quintile, SIMD 5 = least deprived quintile) showing statistically significant difference in proportions across groups (*n* = 428, *X* ^2^ *p* = 0.036).

**Figure S2:** Stackplots of comparing prescription of Angiotensin converting enzyme inhibitors, angiotensin receptor blockers, aspirin and statins by Charlson Comorbidity Index in (A) patients who did undergo surveillance (*p* < 0.001) and (B) patients who did not undergo surveillance (*p* < 0.001) following screening polypectomy in 3534 patients classified as intermediate or high risk by the 2002 British Society of Gastroenterology Guidelines.

## Data Availability

The pseudonymised data set is available on the secure Glasgow Safe Haven TRE platform. Access can be arranged by application to the authors and via the Glasgow SafeHaven TRE following ethical approval and the completion of mandatory information governance modules.
