# Supplementary material for: Retrospective Cohort Study: Scope for Improvement—Barriers to Post‐Polypectomy Surveillance in the Integrated Technologies for Improved Polyp Surveillance Cohort
Source: Aliment Pharmacol Ther. 2025 Jan 20;61(8):1381–6. doi: 10.1111/apt.18514 (PMC11950799; doi:10.1111/apt.18514)
Supplement: Supplementary file 1 — Figure S1: (A) Histogram describing reasons for non‐surveillance in those intermediate/high risk patients not following the 2002 British Society of Gastroenterology Guidelines (n = 1446). (B) Stackplot presenting categorised reasons for non‐surveillance among intermediate/high risk patients in the extremes of Charlson Comorbidity Index (CCI 1–2 = least comorbid, CCI > =5 most comorbid) and Scottish Index of Multiple Deprivation (SIMD 1 = most deprived quintile, SIMD 5 = least deprived quintile) showing statistically significant difference in proportions across groups (n = 428, X 2 p = 0.036). [file APT-61-1381-s002.docx]

**Supplementary Figure 1: A)** Histogram describing reasons for non-surveillance in those intermediate/high risk patients not following the 2002 British Society of Gastroenterology Guidelines (n=1,446). **B)** Stackplot presenting categorised reasons for non-surveillance amongst intermediate/high risk patients in the extremes of Charlson Comorbidity Index (CCI 1-2 = least comorbid, CCI >=5 most comorbid) and Scottish Index of Multiple Deprivation (SIMD 1 = most deprived quintile, SIMD 5 = least deprived quintile) showing statistically significant difference in proportions across groups (n=428, X^2^ p=0.036)

Abbreviations: CCI Charlson Comorbidity Index, NHS GG&C; National Health Service Greater Glasgow & Clyde, SIMD Scottish Index of Multiple Deprivation

**A**

**
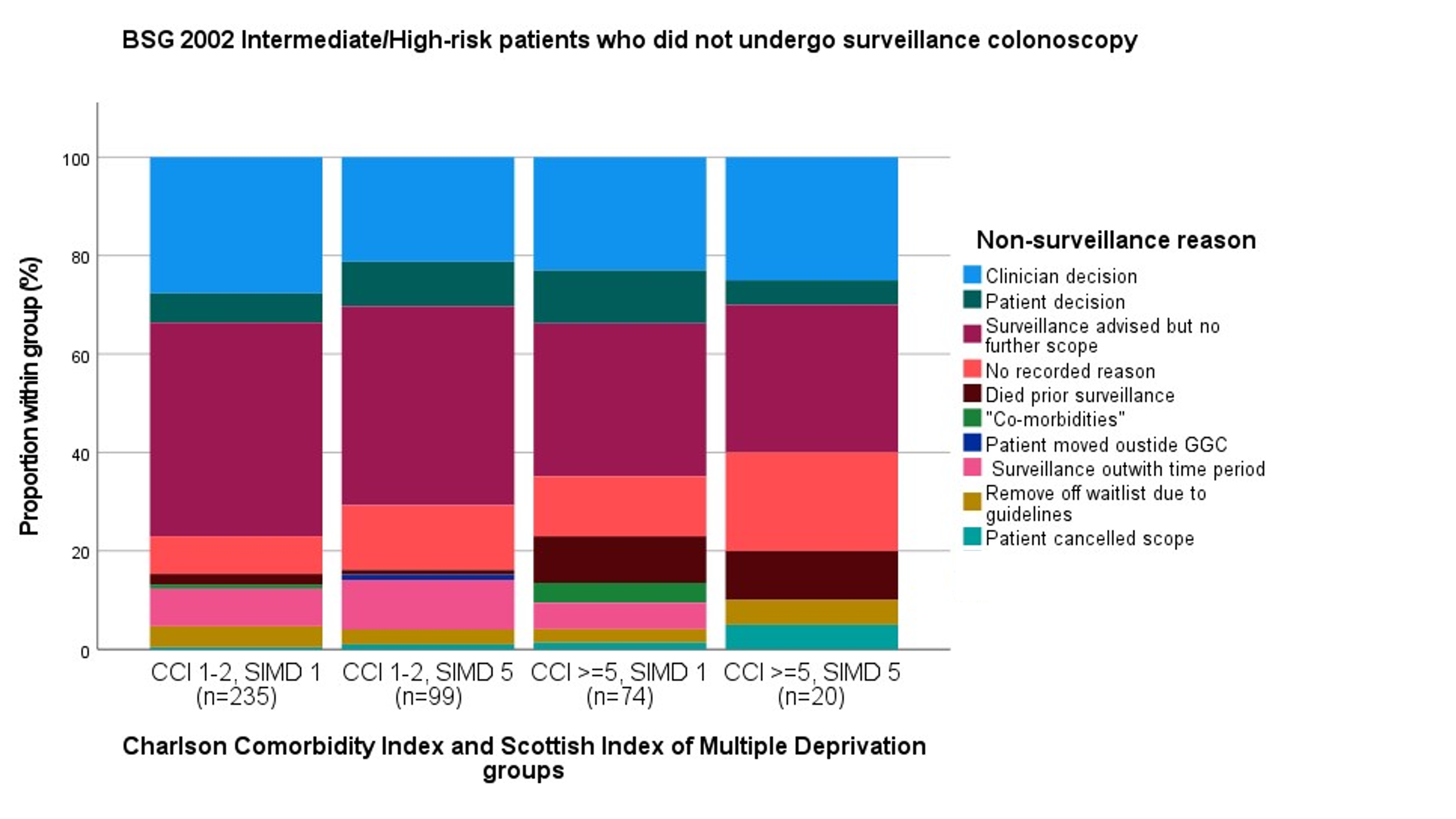

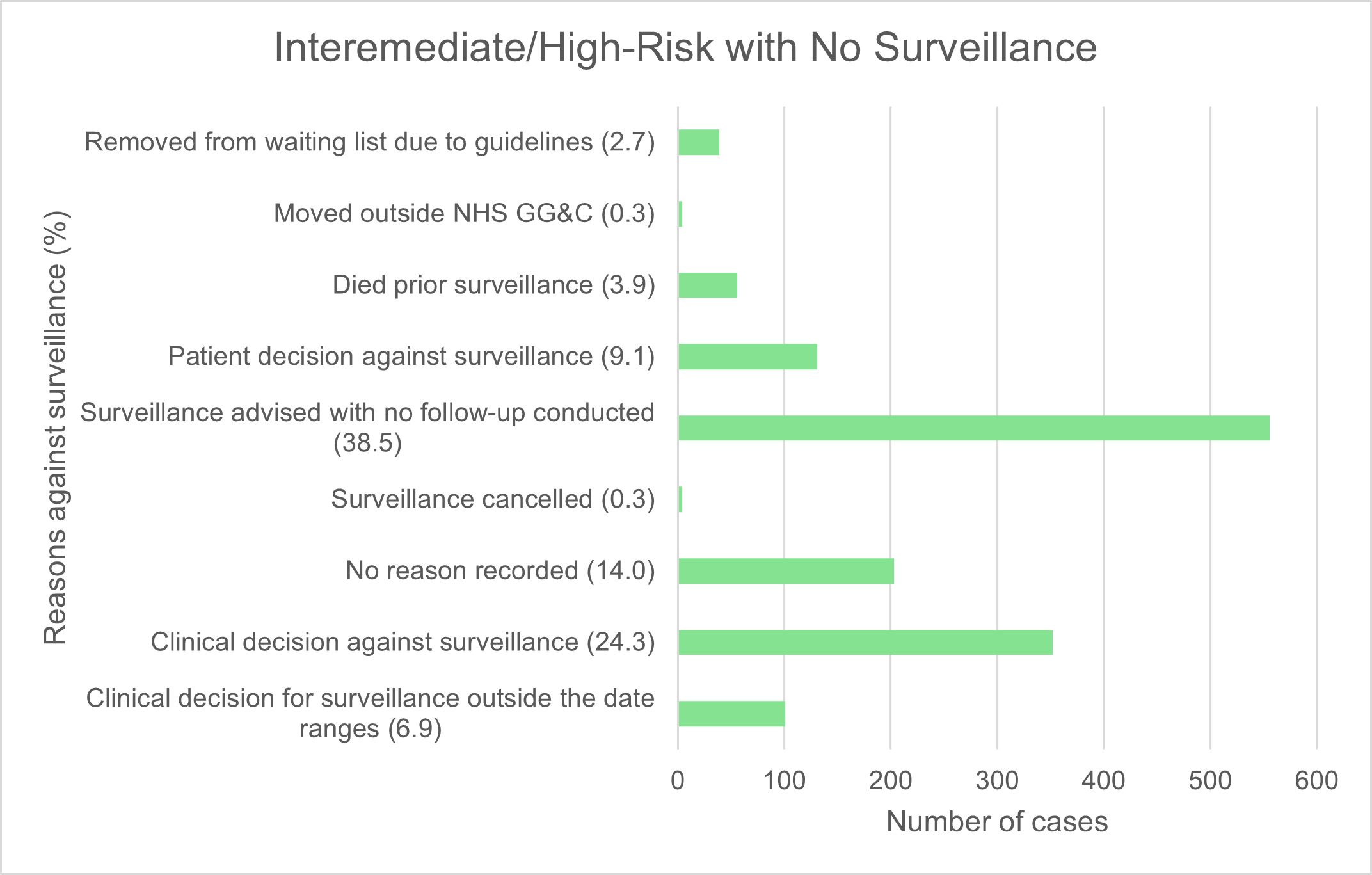
B**
