# Supplementary material for: Retrospective Cohort Study: Scope for Improvement—Barriers to Post‐Polypectomy Surveillance in the Integrated Technologies for Improved Polyp Surveillance Cohort
Source: Aliment Pharmacol Ther. 2025 Jan 20;61(8):1381–6. doi: 10.1111/apt.18514 (PMC11950799; doi:10.1111/apt.18514)

**Supplementary Figure 2:** Stackplots of comparing prescription of Angiotensin converting enzyme inhibitors, angiotensin receptor blockers, aspirin and statins by Charlson Comorbidity Index in **A)** patients who did undergo surveillance (p<0.001) and **B)** patients who did not undergo surveillance (p<0.001) following screening polypectomy in 3534 patients classified as intermediate or high risk by the 2002 British Society of Gastroenterology Guidelines.

Abbreviations: ACE angiotensin converting enzyme, ARB angiotensin receptor blocker, BSG British Society of Gastroenterologists, CCI Charlson Comorbidity Index

**A**


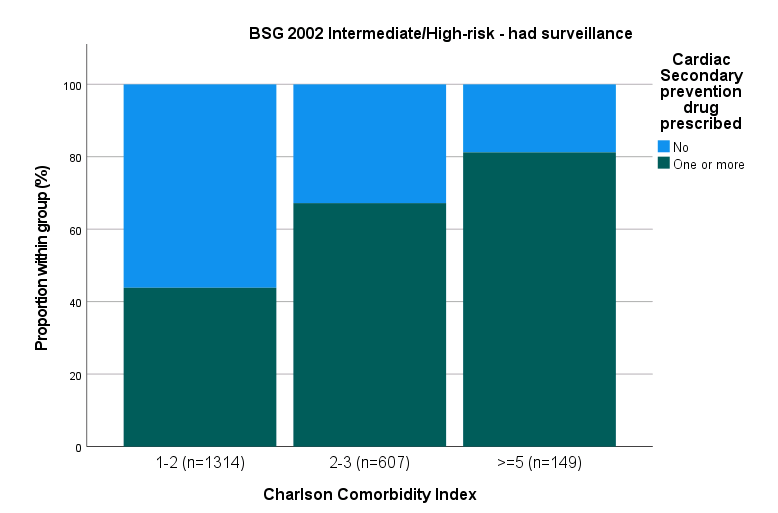


**B**


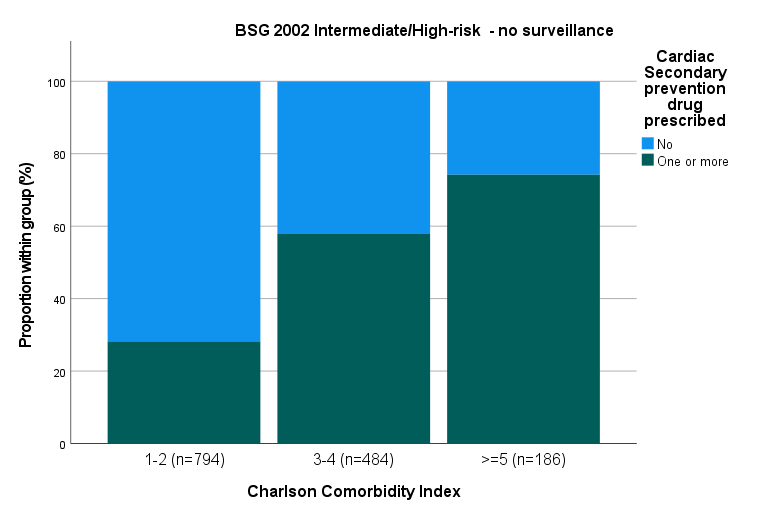

Supplement: Supplementary file 2 — Figure S2: Stackplots of comparing prescription of Angiotensin converting enzyme inhibitors, angiotensin receptor blockers, aspirin and statins by Charlson Comorbidity Index in (A) patients who did undergo surveillance (p < 0.001) and (B) patients who did not undergo surveillance (p < 0.001) following screening polypectomy in 3534 patients classified as intermediate or high risk by the 2002 British Society of Gastroenterology Guidelines. [file APT-61-1381-s001.docx]
